# From Image-Guided Surgery to Computer-Assisted Real-Time Diagnosis with Hyperspectral and Multispectral Imaging: A Systematic Review in Gynecologic Oncology

**DOI:** 10.3390/diagnostics16040620

**Published:** 2026-02-20

**Authors:** Chiara Innocenzi, Matteo Pavone, Barbara Seeliger, Manuel Barberio, Nicolò Bizzarri, Toby Collins, Alexandre Hostettler, Lise Lecointre, Francesco Fanfani, Anna Fagotti, Antonello Forgione, Mariano Eduardo Giménez, Denis Querleu, Jacques Marescaux

**Affiliations:** 1 Dipartimento di Scienze per la Salute Della Donna e del Bambino, UOC Ginecologia Oncologica, Fondazione Policlinico Universitario A. Gemelli IRCCS, Largo Agostino Gemelli 8, 00168 Rome, Italy; 2 Università Cattolica del Sacro Cuore, Largo Francesco Vito 1, 00168 Rome, Italy; 3 Research Institute against Digestive Cancer (IRCAD), 1 Place de l’Hôpital, 67091 Strasbourg Cedex, France; 4 Institute of Image-Guided Surgery, IHU Strasbourg, 1 Place de l’Hôpital, 67091 Strasbourg Cedex, France; 5 ICube, UMR 7357 CNRS, INSERM U1328 RODIN, University of Strasbourg, 67091 Strasbourg Cedex, France; 6 Department of Digestive and Endocrine Surgery, University Hospitals of Strasbourg, 1 Place de l’Hôpital, 67091 Strasbourg Cedex, France; 7 Department of General Surgery, Ospedale Buccheri La Ferla-Fatebenefratelli, Via Messina Marine, 197, 90123 Palermo, Italy; 8 Department of Gynecologic Surgery, University Hospitals of Strasbourg, 1 Place de l’Hôpital, 67091 Strasbourg Cedex, France; 9 DAICIM Foundation, Teaching, Assistance and Research in Minimally Invasive Surgery, Department of General and Minimally Invasive Surgery, University of Buenos Aires, Buenos Aires 1014, Argentina

**Keywords:** hyperspectral imaging, multispectral imaging, image-guided surgery, digital surgery, gynecologic oncology, real-time guidance, artificial intelligence, minimally invasive surgery

## Abstract

**Background:** There is a need for intraoperative image guidance in gynecologic oncologic surgery to provide accurate identification of malignant tissue and ensure negative resection margins. Emerging imaging technologies can complement standard histopathology and reshape intraoperative decision-making. Spectral imaging can extract information on tissue composition and physiological status in real time, without the need for tissue contact, contrast agents, staining, or freezing. This systematic review synthesizes its current clinical applications in gynecologic oncology, decision support utility, and diagnostic performance with data processing frameworks for tissue classification. **Materials and Methods:** This systematic review (PROSPERO: CRD420251032899) adhered to PRISMA guidelines. PubMed, Google Scholar, Embase, ClinicalTrials.gov, and Scopus databases were searched until September 2025. Manuscripts reporting data on spectral imaging in gynecologic oncology were included in the analysis. **Results:** Twenty-nine studies and two clinical trials met the inclusion criteria. Most of them focused on cervical neoplasia (*n* = 17, 58.6%) and ovarian cancer (*n* = 7, 24.1%) detection, followed by assessment of the fallopian tubes (*n* = 2, 6.9%), endometrium (*n* = 1, 3.4%), and vulvar skin (*n* = 2, 6.9%). Using final pathology as the gold standard, overall specificity ranged from 30 to 99%, and overall sensitivity from 75 to 100%, with particularly high sensitivity for cervical lesions (79–100%) and ovarian cancer (81–100%). Among the included studies, thirteen (44.8%) used data interpretation algorithms, of which eleven (84.6%) applied machine learning, one (7.7%) deep learning, and one (7.7%) combined both. **Conclusions:** Spectral imaging, supported by computational methods, has shown promising results in the diagnostic evaluation of gynecologic disease by providing functional and molecular information beyond the capacities of standard visual assessment.

## 1. Introduction

Accurate intraoperative characterization of malignant tissues and the achievement of complete resection with negative margins are central objectives in gynecologic oncology surgery. Real-time histopathological diagnosis in selected cases has a direct impact on decision-making, overall patient management, and clinical outcomes [1,2,3].

The current gold standard of intraoperative frozen section has several constraints: it requires specimen preparation, is time-consuming, and may have suboptimal accuracy, particularly for margin assessment and sentinel lymph node (SLN) evaluation [4]. Although several real-time imaging technologies have emerged, including confocal laser endomicroscopy, optical coherence tomography, optoacoustic imaging, and Raman spectroscopy, most of them still lack sufficient clinical validation for routine intraoperative use [5,6,7]. As a result, there is growing interest in advanced optical instrumentation to provide objective, real-time, and non-invasive assessment of tissue status.

Hyperspectral imaging (HSI) and multispectral imaging (MSI) emerged as safe, non-contact, and non-invasive imaging modalities with significant potential for use in medical diagnostics and intraoperative guidance [8,9]. These technologies exploit the interaction between light and biological tissue to extract a detailed “spectral fingerprint”, which reflects its underlying biochemical and biophysical properties [10]. Preclinical and clinical studies have shown notable results in tumor margin delineation [11], perfusion assessment [12,13,14], anatomical structure differentiation [15], wound evaluation [16], and cancer detection [17,18,19,20].

However, clinical adoption is limited, largely due to the complexity of the spectral data cube, which cannot be immediately interpreted by clinicians. To address this barrier, machine learning (ML), deep learning (DL), and computer-aided diagnostic (CAD) tools became integrated into such imaging workflows to identify and interpret diagnostically relevant spatiospectral features, reduce subjectivity, and facilitate real-time decision support [20,21].

The aim of this systematic review is to outline the diagnostic accuracy and applications of hyperspectral and multispectral imaging in gynecologic oncology. Additionally, it examines existing limitations and discusses how artificial intelligence-driven analytical approaches may help overcome current technical and interpretive challenges to clinical translation.

## 2. Materials and Methods

### 2.1. Search Strategy

This systematic review adhered to the guidelines outlined in the Preferred Reporting Items for Systematic Reviews and Meta-Analyses (PRISMA) [22] and was officially registered with the International Prospective Register of Systematic Reviews (PROSPERO: CRD420251032899) before data extraction. A systematic search for “hyperspectral imaging”, “multispectral imaging”, “spectral imaging”, “optical imaging”, “gynecology”, “gynecologic oncology”, and “gynecological neoplasm”, filtered to include only English-language content, was conducted using PubMed, Google Scholar, Embase, ClinicalTrials.gov, and Scopus databases until September 2025, with the complete search strategy reported in Supplementary Materials (File S1).

### 2.2. Data Extraction and Analyses

Rayyan software (https://www.rayyan.ai/, accessed on 1 April 2025, web-based SaaS, Qatar Computing Research Institute, HBKU, Doha, Qatar) [23] was used independently by two authors (CI and MP) to screen titles and abstracts for eligibility. After removal of duplicate publications, keywords and full texts were independently reviewed for inclusion. PICO criteria were defined as follows:

Population (P): Studies were eligible if they involved patients undergoing diagnostic or therapeutic procedures for gynecologic malignancies in which HSI or MSI technologies were used. Only clinical studies were included in the analysis; animal models or other preclinical studies were excluded.

Intervention (I): The intervention of interest was the use of HSI or MSI for real-time high-resolution tissue characterization during diagnostic assessment or surgical procedures, with all available platforms, including both in vivo applications and ex vivo analyses of freshly excised specimens.

Comparison (C): Studies comparing the diagnostic performance of HSI/MSI technologies with the diagnostic gold standard of conventional histopathological examination were included.

Outcome (O): The primary outcomes of interest were diagnostic performance metrics of HSI/MSI, including sensitivity, specificity, positive predictive value (PPV), negative predictive value (NPV), and accuracy. Secondary outcomes included the integration with digital technologies (e.g., artificial intelligence algorithms).

Prospective and retrospective original clinical research articles, published in English, were included. Conference abstracts, systematic reviews, meta-analyses, editorials, and letters to the editor were excluded.

For each included study, the following data were systematically extracted: year and country of publication, study design, sample size, analyzed target tissue, modality of HSI/MSI used (in vivo, ex vivo, or both), technical specifications of the imaging system and scan method, acquisition time, reported values for sensitivity, specificity, PPV, NPV, and accuracy. When available, data on the integration of artificial intelligence (AI) with ML and DL models or other digital tools were also collected. Additionally, each study was classified according to its IDEAL stage, reflecting the technological level of maturity and validation [24].

### 2.3. Risk of Bias

The methodological quality of studies assessing diagnostic accuracy was independently evaluated by two reviewers (CI and MP) using the Quality Assessment of Diagnostic Accuracy Studies 2 (QUADAS-2) tool [25]. Studies reporting sensitivity, specificity, or other measurements of diagnostic performance were included in this assessment. Bias was assessed across four domains, namely patient selection, index test, reference standard, and flow and timing.

### 2.4. Spectral Imaging: Technological Principles and Medical Applications

Conventional imaging systems use the RGB (Red–Green–Blue) color representation, in which each pixel encodes the intensity of three primary visible light bands: red (622–780 nm), green (492–577 nm), and blue (455–492 nm), limited to the visible spectrum. Light propagation in biological tissues involves refraction, scattering, absorption, and fluorescence, four photophysical processes that alter the speed, direction, and spectral composition of light, beyond the visible spectrum [26]. Such light–tissue interactions produce a unique spectral and spatial signature across the electromagnetic spectrum, often referred to as a “fingerprint”. These fingerprints can be captured using multispectral and hyperspectral imaging systems, which sample wavelength-dependent information. These images require a broadband spectrum of electromagnetic waves acquired for every single pixel. This process results in a three-dimensional dataset, namely the *hypercube*, comprising two spatial dimensions (x, y) and one spectral dimension (z) [26].

**Figure 1:**
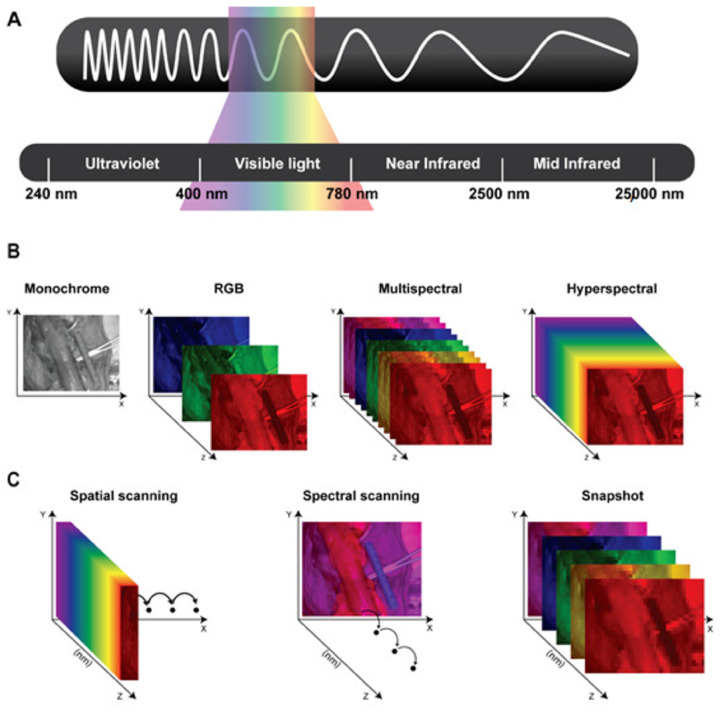
(**A**) Schematic representation of the electromagnetic spectrum’s wavelengths: Ultraviolet (240–400 nanometers), Visible light (400–780 nanometers), Near-infrared (780–2500 nanometers), and Mid-infrared (2500–25,000 nanometers). (**B**) Representation of the different datasets generated using monochrome images; color or RGB (red, green, and blue) images; and multispectral and hyperspectral imaging. (**C**) Schematic representing three different types of hyperspectral imaging devices. Spatial scanning: acquiring the spectral information as a whole and progressively scanning the spatial information. Spectral scanning: acquiring the spatial information as a whole and scanning the spectral information. Snapshot: simultaneously acquiring spectral and spatial information, but providing a lower spatial and spectral resolution than previously mentioned hyperspectral imagers. Courtesy of [8].

By analyzing the hypercube across multiple wavelengths and using digital imaging integrated with optical spectroscopy, HSI and MSI systems provide rich information beyond the visible spectrum and enable the extraction of biochemical and morphological information at a molecular level. HSI systems generally generate images with a resolution of over several narrow (e.g., at 10 nm intervals) bands, spanning a wide spectral range, typically from ultraviolet (UV) to near-infrared (NIR). In the literature, the distinction between multispectral and hyperspectral imaging is not governed by a universally accepted numerical threshold [27]. While multispectral systems in remote sensing may comprise a larger number of spectral bands, several surgical imaging publications adopt practical thresholds in the order of 10 bands for conceptual clarity, with discrete or (near-)continuous spectrum measurement, and we follow this convention to reflect prevailing usage [28,29] (Figure 1).

Consequently, spectral imaging provides both qualitative and quantitative assessment of tissue composition through distinct spectral signatures arising from light absorption in a non-invasive, non-contact, non-ionizing, and label-free sensing method.

The fundamental theory behind the clinical use of HSI/MSI is that pathological and healthy tissues exhibit different spectral signatures. These can be leveraged for diagnostic purposes, such as quantifying hemoglobin and deoxyhemoglobin concentrations to infer tissue perfusion and angiogenesis, as well as elevated metabolic activity and water, lipid, and protein content, which yield measurable spectral differences relative to normal tissue [10].

Concerning medical applications, HSI and MSI are generally classified according to their acquisition mode into three primary scanning device categories, each with distinct trade-offs in spectral and temporal resolution, namely spatial scanning, spectral scanning, and snapshot (Figure 1) [30]. Spatial scanning acquisitions capture high-resolution spectral information as a whole while progressively scanning the spatial information (pixel by pixel: point scanning/whiskbroom; line by line: line-scanning/push-broom), limited by the resolution and speed of the spatial scanning process of several seconds and susceptibility to motion artifacts. Spectral scanning acquisitions acquire high-resolution spatial information as a whole, while the spectral coverage depends on the (tunable) filters used, limited by the spatial scanning process for each wavelength and susceptibility to motion artifacts. Snapshot devices enable real-time imaging at video rate by capturing spectral and spatial data simultaneously, albeit at the cost of reduced spatial and spectral resolution [8,14].

## 3. Results

### 3.1. Study Characteristics

**Figure 2:**
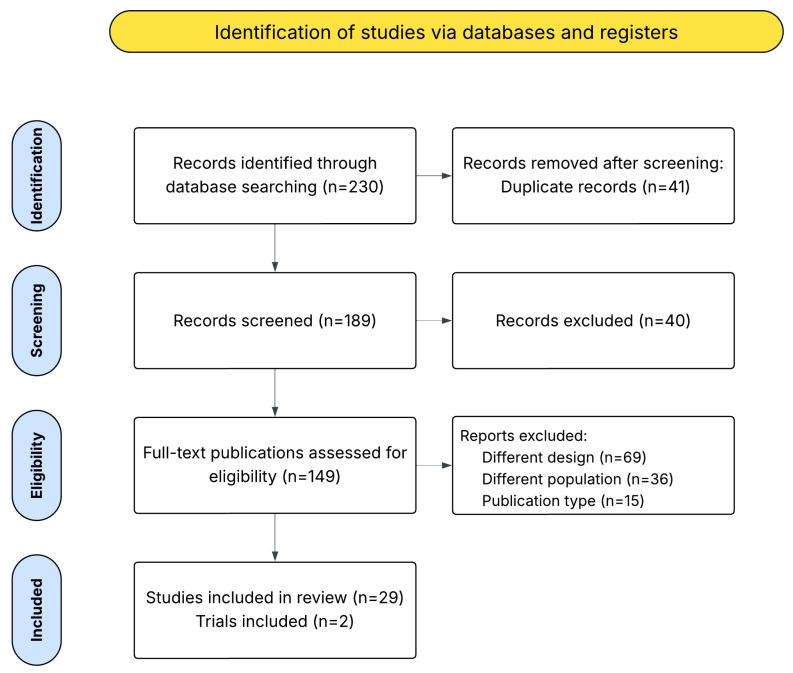
PRISMA flow diagram of selected studies.

The search strategy identified 230 articles, including 29 studies and 2 clinical trials, which met the inclusion criteria for the systematic review (Figure 2, Table S1). Table S2 in Supplementary Materials summarizes the characteristics of the included studies, IDEAL stage, sample size, camera types, scanning methods, examined tissue types, wavelength ranges, and—when available—the AI models used. Due to the scarcity of publications, the analysis remained qualitative. Excluded articles are listed in Supplementary Materials (Table S3).

Most investigations focused on cervical neoplasia (*n* = 17, 58.6%) and ovarian cancer (*n* = 7, 24.1%) detection, followed by studies on fallopian tube evaluation (*n* = 2, 6.9%), endometrium assessment (*n* = 1, 3.4%), and vulvar skin evaluation (*n* = 2, 6.9%). All studies were prospective in design. Specifically, 17 (58.6%) of the 29 studies used multispectral technology, whereas 12 (41.4%) used hyperspectral imaging; the corresponding spectral ranges, which differ based on the camera type used, are reported in Table S2. Regarding the application mode, 21 studies (72.4%) used spectral imaging modalities in vivo, 6 studies (20.6%) used ex vivo, and 2 studies (6.8%) used both modalities. Two ongoing trials were identified from the literature search, with a total cohort of 1018 patients (Supplementary Materials Table S4), and both involve the use of spectral imaging to detect cervical neoplasia.

### 3.2. Risk of Bias

QUADAS-2 was applied to all studies reporting any evaluable diagnostic performance measurements. However, four studies could not be assessed with QUADAS-2 as they focused on feasibility or technical development rather than diagnostic accuracy outcomes [31,32,33,34].

Although most studies were prospective, overall, the QUADAS-2 assessment indicated a moderate-to-high risk of bias, with the most frequent limitations affecting patient selection and index test domains. In contrast, the reference standard and flow/timing domains were generally more robust. Applicability concerns were mainly driven by patient selection and index test domains, suggesting that results may not be fully generalized to intended clinical screening or triage populations (Supplementary Materials Table S5).

### 3.3. Diagnostic Performance

Diagnostic performance was evaluated against histopathological examination. However, sensitivity and specificity relative to definitive pathological diagnosis were not consistently reported across the included studies (Table 1). When aggregating data across all lesion types, overall diagnostic performance metrics (sensitivity, specificity, and accuracy) were reported in twelve studies (41.4%). Reported sensitivities ranged from 75 to 100%, whereas specificities ranged from 30 to 99%. Sensitivity was reported in twelve (41.3%) studies, with eight (66.6%) of these reporting values exceeding 90%. Specificity was reported in eleven (37.9%) studies, of which five (45.4%) presented values higher than 85%.

Four (33.3%) studies focused on detecting ovarian carcinoma reported sensitivity values ranging from 81 to 100% and specificity values ranging from 69 to 93.5% [40,42,43,45].

All studies on ovarian cancer detection used histopathological analysis as the gold standard. However, they were heterogeneous with respect to patient and tissue characteristics. Notably, some studies did not distinguish between patients undergoing primary debulking surgery versus interval debulking surgery, and therefore between chemotherapy-naïve or previously treated tumor tissues. Additionally, no subgroup analyses according to tumor histology were performed, although most cases consisted of serous epithelial ovarian cancer.

**Figure 3:**
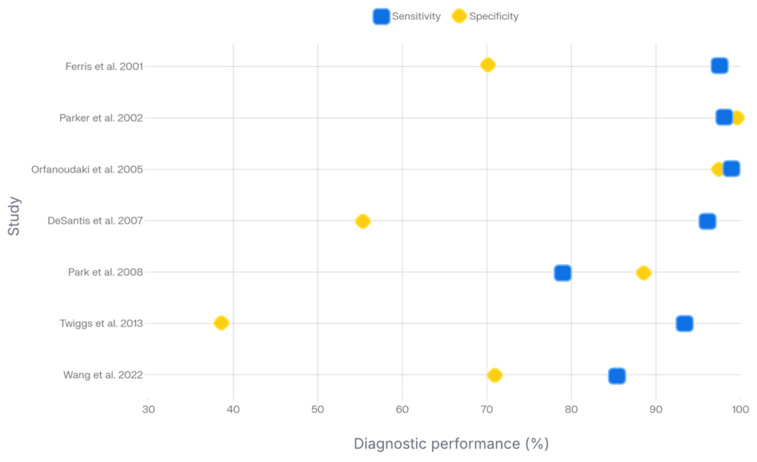
Diagram of diagnostic performances of hyperspectral and multispectral imaging for cervical preneoplastic and neoplastic lesions [35,36,37,38,39,41,44].

Seven (58.3%) studies investigated the differential diagnosis of cervical lesions, reporting overall sensitivity ranging from 79 to 100% and specificity ranging from 30.3 to 98.9% (Figure 3) [35,36,37,38,39,41,44]. Of these studies, six used histopathological analysis as the reference standard. Most studies focused on lesions ranging from CIN I to CIN III using spectral imaging to distinguish them from normal tissue [35,36,37,38,41], whereas one preliminary study evaluated the ability to discriminate cancerous areas from healthy tissue [44].

One study (8.3%), which evaluated the feasibility of HSI for assessing therapeutic response in vulvar lichen sclerosis, reported a sensitivity of 75% and a specificity of 87.5% [32].

A total of 10 studies reported image acquisition time. However, there was considerable variability, largely due to the inclusion of different subtypes of spectral imaging techniques.

Most studies were in the early stages of surgical innovation. According to the IDEAL framework, some were proof-of-concept studies (stage I; 7/29, 24.1%), and the majority were developmental or exploratory (stages IIa–IIb; 22/29, 75.9%). None reached randomized controlled assessment of effectiveness against current standards (stage III), or long-term monitoring and registry (stage IV).

### 3.4. Artificial Intelligence Integration

Some studies investigated the early implementation of AI to support or enhance diagnostic interpretation. Thirteen studies (44.8%) used AI-based algorithms, whereas sixteen (55.2%) did not. Of those using AI, eleven (84.6%) applied classical machine learning (ML), one (7.7%) used deep learning (DL), and one (7.7%) combined both.

Among the classical ML models, the most frequent ones were unsupervised k-means clustering (3/11, 27.3%) and linear discriminant analysis (LDA) (3/11, 27.3%), followed by support vector machines (SVM) (2/11, 18.2%) and other supervised classifiers such as logistic regression, k-nearest neighbors, random forest (RF), and ensemble models. The DL approaches described in the two relevant studies used convolutional neural networks (CNNs).

One study reported that LDA outperformed other tested models, including SVM, k-means clustering, and spectral angle mapping (SAM), which showed a lower accuracy, partly ascribable to the unbalanced and relatively small dataset, as acknowledged by the authors [32]. To enable reliable implementation and clinical translation of such models, larger and more representative datasets will be required. Another study compared the performance of a random forest (RF) classifier with that of a convolutional neural network (CNN) [46]; the CNN achieved higher tissue type segmentation accuracy, likely due to its ability to use a broader range of information [46].

## 4. Discussion

Spectral imaging technologies are emerging as *optical biopsy* methods with promising diagnostic capabilities in the field of gynecologic oncology, particularly for the detection of cervical and ovarian neoplastic lesions. This systematic review found high sensitivities for cervical lesions (79–100%) and ovarian cancer (81–100%). The variability of image acquisition times reflected the variety of spectral imaging systems and acquisition protocols. A progressive integration of computer assistance to facilitate tissue classification and lesion detection was identified, with 44.8% of studies applying classical ML or DL models.

These technologies hold considerable diagnostic potential for malignancy detection, as the neoplastic transformation alters tissue morphological, biochemical, and optical properties (such as absorption, scattering, and fluorescence), providing targets for advanced biophotonics imaging technologies [26,38]. According to the IDEAL framework, the included studies predominantly correspond to the early stages of surgical innovation, indicating limited technological maturity and clinical validation. In biomedical applications, medical devices can also be classified according to their Technology Readiness Level (TRL), being validated in preclinical and feasibility studies (TRL 4 to 5), demonstrated in early clinical studies (TRL 6), assessed in late clinical studies and evidence building (TRL 7–8), or undergoing commercialization (TRL 9) [47,48,49]. Accordingly, the spectral imaging devices evaluated in this systematic review ranged from TRL 4 to 7 when applied to gynecologic oncology, with limited large-scale clinical validation or routine deployment. Overall, the current level of evidence is based on the early stages of innovation, relatively small cohorts, and methodological heterogeneity.

### 4.1. Cervical Neoplasia Screening and Diagnosis

The standard approach for diagnosing preneoplastic cervical lesions involves cytology and colposcopy combined with targeted cervical biopsies [50]. However, this method is inherently subjective and highly dependent on the clinician’s experience, leading to considerable inter- and intra-observer variability and limited diagnostic accuracy, with sensitivity ranging from 68.5 to 77% and specificity from 75.9 to 82%, frequently resulting in biopsies that could be avoided [39,41,44].

HSI and MSI can detect atypical vascular patterns and analyze in vivo fluorescence and reflectance spectra from the cervix [34]. Through derivative spectral analysis, key diagnostic wavelengths can be identified, allowing real-time tissue classification into normal, inflammatory, and high-grade lesions, without the need for biopsy [51,52,53,54]. In comparative analyses, HSI demonstrated a sensitivity of 97% for detecting high-grade cervical lesions (CIN II or greater), outperforming the 72% sensitivity of the Papanicolaou test (Pap smear), with an equal specificity of 70% [35,41]. Similarly, the integration of HSI into colposcopy achieved 95% sensitivity for CIN II+ detection, with a corresponding specificity of 55% for benign cervices [38], whereas multispectral digital colposcopy achieved 79% sensitivity and 88% specificity [39]. The 555 to 585 nm hyperspectral range was shown to discriminate cervical intraepithelial neoplasia from healthy tissue, with the highest accuracy at 575 nm (*p* = 0.0048) [55].

When synergized with AI, optical imaging technologies represent a powerful innovation in cervical cancer diagnostics [36,44,46,51,56,57]. For instance, AI-based multispectral imaging achieved 85.3% sensitivity and 70.8% specificity in differentiating between pathological and normal cervical tissue, outperforming unassisted imaging techniques [44].

Additionally, studies have investigated the effects of acetic acid on tissue properties, demonstrating that its application alters the mean light scattering coefficient of precancerous tissue. MSI colposcopy may capture distinct temporal patterns in tissue response following acetic acid application, allowing differentiation between various lesion types [58]. For instance, CIN lesions exhibited distinctive temporal signatures, with higher-grade lesions showing prolonged whitening effects (>500 s for CIN III vs. ~200 s for CIN II) [59]. In addition, the shape of the IBSL curve (intensity of the backscattered light) and the relaxation time (the interval required for the IBSL signal to decay to 1/e of its peak value) were reported to vary according to the underlying lesion. Notably, MSI colposcopy demonstrated a remarkably low false-diagnostic rate of 1.7% (misclassification or false-negative results), as compared to 24.4% for Pap smear tests and 22% for conventional colposcopy [37]. In an innovative pilot study, a portable, low-cost, label-free transvaginal multispectral probe was developed, and the spectral contrast ratio (ρ) at 450 nm and 545 nm was found to be increased in premalignant tissues compared to normal tissues [60].

Although these results are encouraging, several limitations persist. The influence of blood, cervical mucus, or active infections on spectral signal interpretation has not been fully elucidated and warrants further investigation [35,60]. Nevertheless, after rigorous validation, these technologies could guide targeted biopsy sampling, providing valuable support for clinicians with varying levels of expertise.

### 4.2. Vulvar Disease Assessment and Monitoring

Building on the same optical principles, polarized hyperspectral imaging has been applied to the assessment of vulvar pathologies as a non-invasive diagnostic tool for in vivo detection of vulvar lichen sclerosis, by capturing subtle alterations in tissue structure and composition [61]. In a preliminary study, HSI and active dynamic thermal (ADT) imaging were applied to evaluate treatment response after high-intensity focused ultrasound (HIFU) therapy in patients with lichen sclerosis, for therapeutic monitoring and the early identification of non-responders. ADT demonstrated superior performance, achieving 100% sensitivity and specificity, whereas HSI reached 75% sensitivity and 87.5% specificity [32]. The authors attributed this difference to the HSI imager’s spectral range (400–700 nm), suggesting that additional spectral information with a broader wavelength coverage, such as 400–1000 nm, could improve diagnostic accuracy. Treatment response in this study was assessed subjectively by a gynecologist using a clinical scoring method, which might have introduced variability and limited the objectivity of the evaluation.

### 4.3. Ovarian Cancer Detection

A multispectral spatial frequency domain imaging (SFDI) system was used for ex vivo assessment of human ovarian tissue. Fourteen ovaries were imaged and, using a logistic regression model, the system achieved a sensitivity of 94.06%, a specificity of 93.53%, a PPV of 92.23%, and an NPV of 95.04% for the prediction of histologically confirmed invasive carcinoma. Malignant ovaries exhibited a significantly higher total hemoglobin concentration and reduced scattering amplitude and slope, consistent with increased angiogenesis and decreased stromal collagen, as confirmed by histological analysis [42]. Additionally, a multispectral imager was used to acquire autofluorescence images across multiple spectral bands from various ovarian tissue samples, achieving a sensitivity of 100% and a specificity of 51.1% in differentiating normal from malignant ovaries [40]. Of note, a key limitation identified was the presence of spatial heterogeneity in tissue optical properties, driven by hypervascular regions and surface blood in the region of interest (ROI), which introduced major confounding effects. Specificity was improved to 68.5% without compromising sensitivity by normalizing autofluorescence data using a reflectance-based division method. As a result, correcting for optical heterogeneity in tissue imaging is key using such technologies [40]. However, specificity remained suboptimal, as endometriomas were frequently misclassified as malignant, even after applying the correction.

Several authors have acknowledged the technical limitations inherent to current spectral imaging systems designed for superficial tissue assessment and their limited capability to detect small foci of deep-seated invasion, such as those occurring within large ovarian masses [42,43]. In this context, co-registered multispectral photoacoustic tomography (PAT) combined with pulse-echo ultrasound (US) was investigated as a functional in vivo imaging approach for ovarian masses characterization [62]. Photoacoustic imaging is a hybrid optoacoustic modality that detects acoustic signals generated by thermoelastic expansion after pulsed laser excitation from a few millimeters to centimeters in depth [63]. In a pilot in vivo multispectral PAT study, invasive epithelial ovarian cancer was shown to be associated with higher tumor vascularity, increased total hemoglobin concentration, and lower oxygen saturation as compared to benign or normal ovaries [62].

Patient survival in ovarian cancer is strongly dependent on the completeness of cytoreductive surgery as a major independent prognostic factor [64]. The use of AI is increasingly being explored not only to improve diagnosis through radiomics-based analysis, but also through computer vision applied to laparoscopic images to predict treatment outcomes, simplify triage, and improve early treatment planning by stratifying the patients at the diagnostic laparoscopy stage [65]. Accordingly, the development of imaging techniques capable of intraoperatively detecting microscopic tumor deposits would add significant clinical value. A pilot study demonstrated the feasibility of HSI combined with an ML classification model for detecting epithelial ovarian cancer (EOC) in ex vivo tissue samples from patients undergoing primary or interval cytoreductive surgery. A linear SVM model achieved an area under the curve (AUC) of 0.83, a sensitivity of 0.81, a specificity of 0.70, and a Matthews correlation coefficient of 0.41 in tumor tissue classification [43]. However, discrepancies may arise between the imaging depth of the hyperspectral camera and the histopathological reference standard, as well as from potential misregistration between HS images and corresponding histological slides, due to tissue deformation during specimen handling and processing [43]. Likewise, an ML model applied to multispectral Raman spectroscopy for the detection of ovarian and endometrial cancer achieved a 93% sensitivity analyzing only eight spectral bands, despite the limited size of the training dataset [45]. However, future analyses should account for additional clinical factors, such as prior neoadjuvant chemotherapy, which may influence the tissue’s optical properties, and specific biomarkers. In ovarian cancer, folate receptor alpha (FRα) is overexpressed in 92 to 97% of malignant tumors, especially serous carcinomas [66]. Tumor-specific multispectral fluorescence imaging using FRα-targeted fluorescent agent provides sensitive real-time identification of tumor tissues during staging and debulking procedures in patients with FRα-positive ovarian cancer. Promising results both for in vivo and ex vivo imaging support the potential of tumor-specific fluorescent tracers to further expand this approach [67].

### 4.4. Fallopian Tubes (FT) and Endometrium Analysis

A novel instrument, called a falloposcope, has recently been proposed as it integrates optical coherence tomography (OCT) and multispectral imaging [68]. It was initially developed for in vivo visualization of the salpingeal lumen, considered the site of origin of most high-grade serous ovarian cancer [68]. In a feasibility study, the device was safely inserted under hysteroscopic guidance and advanced into the FTs during a 15 min pause in standard-of-care surgery, enabling high-resolution imaging of the tubal epithelium. Although navigating the narrow tortuous proximal FT segment posed technical challenges, the study demonstrated the safety and potential of this approach for early detection and surveillance of tubal abnormalities [31,33]. OCT has increasingly been adopted across several medical specialties, including oncologic surgery, due to its ability to generate high-resolution histology-like images within minutes and without the need for contrast agents or staining. Reported diagnostic performance has reached up to a 96% accuracy across a range of cancer types [6]. An endoscopic spectral imaging system integrating an ML algorithm enabled in vivo multispectral analysis of the endometrium, identifying five spectral clusters that correlate with tissue pathology [69]. A long-term goal may be the integration of these technologies into laparoscopic procedures, enabling in vivo diagnostics and real-time screening for patients with an elevated risk of developing cancer, as well as supporting fertility-preserving treatment strategies [70].

### 4.5. Artificial Intelligence and Spectral Imaging

The clinical use of spectral imaging in gynecologic oncology remains limited due to several practical challenges, such as acquiring images with high spatial and spectral resolution, maintaining a high signal-to-noise ratio (SNR), and interpreting the resulting data. In addition, trade-offs exist among these key parameters across different hyperspectral sensor systems, making it difficult for users to take full advantage of all their features [71]. With advancements in computer science, various AI algorithms have been used for the classification and identification of spectral images [71].

In this context, pixel-wise algorithmic analysis of the hypercube can effectively decipher the complex spectrospatial information for medical imaging applications such as blood perfusion and ischemia monitoring, wound vascularization and inflammation, tissue differentiation, and tumor detection [14].

Among gynecologic oncology studies, ML algorithms predominate. From a clinical perspective, standardized imaging protocols, robust automated segmentation tools, and large, well-annotated datasets are essential for the effective training of these models.

To overcome data limitations, expand the training set, and improve performance, hypercubes from multiple cancer types may be combined to train models that distinguish between healthy and cancerous tissue, irrespective of any specific tumor origin. Additionally, adjusting classification thresholds on a per-patient basis significantly enhanced accuracy as compared to fixed thresholds [20]. This approach has been explored in gastrointestinal cancers, whereas current AI models in gynecologic oncology remain tailored to specific malignancies, limiting generalizability.

A potential limitation in studies involving small and heterogeneous datasets lies in the risk that the model may learn spurious correlations—for instance, associating diagnostic labels with spectral features unrelated to the underlying biomolecular factors of interest, such as the presence of cancer cells and their associated biochemical or structural changes [45]. Such biases typically arise from unbalanced datasets or limited sampling across tissue types, leading to overfitting and poor generalizability. To overcome such issues, it is essential to ensure a balanced and representative dataset that includes both normal/benign and malignant spectra from multiple patients and organ sites, thereby minimizing confounding factors and enhancing the robustness and translational reliability of the model.

In the era of digital healthcare, AI models are increasingly recognized for their potential as decision-support tools to enhance the clinician’s performance [72]. However, these models often operate as “black boxes,” limiting the transparency of their decision-making processes and undermining the confidence of the users in their predictions. This concern has stimulated the creation of *Explainable Artificial Intelligence* (XAI), in which algorithms elucidate which features or patterns most strongly influence the outputs [73]. The need for explainability is particularly relevant in the context of medical devices, where AI-driven decisions must meet stringent requirements of reliability, safety, and clinical trustworthiness. Accordingly, before deploying AI models for the interpretation of spectral imaging data in clinical settings, explainability techniques should be applied to verify that model decisions are biologically and clinically plausible, to ensure that the best-performing and most reliable model is selected for production, and to confirm that the system is functioning as intended.

### 4.6. Strengths and Limitations

This systematic review provides a comprehensive synthesis of the current clinical applications and diagnostic accuracy of hyperspectral and multispectral technologies in gynecologic oncology. Its notable strengths include adherence to a registered PROSPERO protocol, a rigorous study selection process, compliance with PRISMA guidelines [22], the reporting of diagnostic performance indicators, and IDEAL stages of innovation. The limited number of eligible studies and the heterogeneity of available data did not allow for a quantitative meta-analysis. The nomenclature distinguishing between HSI and MSI at varying thresholds of the number of spectral bands involved is heterogeneous, and terms such as “optical imaging” are used ambiguously. The use of custom-built or prototype systems in early clinical assessment stages implied a modest standardization in image acquisition and interpretation. Accordingly, there was heterogeneity in terms of study methodology, design, sample size, and outcome reporting. Few spectral imaging systems are currently approved in surgical settings and commercially available [30]. In the field of gynecologic oncology, studies carried a high risk of bias due to non-randomized designs, unclear blinding and index test assessment, and the lack of external validation of decision support algorithms. For generalizability and broad clinical adoption, there remains a need for advanced validation studies, including correlations with clinical outcomes.

### 4.7. Future Perspectives

Spectral imaging has the potential to extend surgeons’ vision at the molecular and cellular levels. Recent pilot studies on freshly excised colorectal and esophageal cancer specimens have demonstrated that HSI, when combined with ML algorithms, can accurately differentiate malignant from healthy tissue [74]. In gynecologic oncology, there are promising results in tumor identification, particularly for ovarian cancer and cervical pathologies.

However, diagnostic sensitivity and specificity of spectral imaging exhibit considerable variability, attributable to methodological heterogeneity across studies, including variations in imaging systems, spectral ranges, acquisition protocols (in vivo vs. ex vivo), and small, unbalanced datasets. These limitations undermine generalizability to multi-center settings, underscoring the need for protocol harmonization and external validation on larger representative cohorts.

Beyond tumor identification, HSI can monitor intraoperative tissue oxygenation, as demonstrated in ischemia models and applications in liver surgery, to distinguish perfused from non-perfused areas and guide resection [75]. Additionally, acting as a theranostic tool, HSI can assess thermal damage during laser ablation therapies, predicting thermal thresholds and the extent of necrosis [76]. However, such applications remain unexplored in gynecologic oncology, and evidence is limited to diagnostic applications. Overall, the integration of spectral imaging with AI to enhance precision in surgical settings marks a step towards the emerging field of *surgical optomics* [15].

Although several systems are currently available for open surgery, ongoing efforts aim to miniaturize these technologies for minimally invasive procedures without compromising spectral and spatial resolution [14,73]. A long-term objective is to integrate miniaturized spectral sensors for direct intraoperative application, allowing seamless incorporation with endoscopic and laparoscopic instrumentation, and addressing challenges related to uncalibrated illumination [77].

Concurrently, the development of multimodal platforms that combine spectral imaging with complementary optical modalities aims to personalize surgical interventions based on the specific pathology and individual patient characteristics [78]. Despite challenges related to data processing, standardization, and system complexity, the future of spectral imaging in image-guided surgery is promising [79]. Advances in integrated systems, multiparametric analysis, and AI foster the incorporation of spectral imaging into the intraoperative workflow, supporting the emerging concept of the *smart hybrid operating room*.

## 5. Conclusions

The optimization of gynecologic oncology patient management mandates the development of more rapid and reliable diagnostic technologies capable of early detection and precise intraoperative assessment. Within the contemporary framework of precision medicine and digital surgery, spectral imaging offers a rapid, non-destructive analytical approach that provides detailed insights into the molecular and biological composition of tissues. Despite the heterogeneity of devices and studies limiting the generalizability of the reported accuracy metrics, pooled evidence reports consistently high diagnostic sensitivity, particularly for cervical and ovarian malignancies. To fully realize the clinical potential of spectral imaging, future studies should prioritize methodological harmonization, multicenter validation of imaging sequences and metadata, correlation with clinically meaningful endpoints to substantiate the diagnostic value, and clinical applicability of such emerging technologies.

## Figures and Tables

**Table 1:** Hyperspectral and multispectral sensitivity, specificity, and accuracy for gynecologic malignancies (abbreviations: CIN, cervical intraepithelial neoplasia; NPV, negative predictive value; PPV, positive predictive value).

Year	Author	Trial Design	Sample Size	Target Tissue	Optical Type	Sensitivity (%)	Specificity (%)	PPV (%)	NPV (%)	Accuracy (%)
2001	Ferris DG et al. [35]	Phase I	111	Cervix	Hyperspectral	97%	70%	/	/	/
2002	Parker MF et al. [36]	Phase I	33	Cervix	Hyperspectral	98.2%	98.9%	71.4%	99.9%	97.50%
2005	Orfanoudaki IM et al. [37]	Phase I	134	Cervix	Multispectral	Overall: 98.3% CIN II+: 100%	CIN II+: 98.1%	CIN II+: 90.0%	CIN II+: 100%	/
2007	DeSantis T et al. [38]	Phase II	572	Cervix	Hyperspectral	CIN I: 78.8% CIN II+: 95.5%	55.2% for benign lesions	/	/	/
2008	Young Park S et al. [39]	Pilot	29	Cervix	Multispectral	79%	88%	/	/	/
2012	Renkoski TE et al. [40]	Phase II	30	Ovaries	Multispectral	100%	69%	/	/	/
2013	Twiggs LB et al. [41]	Pilot	1607	Cervix	Hyperspectral	CIN II: 91.3% CIN III: 93%	Normal: 38.9% CIN I: 30.3%	CIN I: 24.7%	CIN I: 94.4% CIN III: 99%	/
2018	Nandy S et al. [42]	Pilot	11	Ovaries	Multispectral	94.06%	93.53%	92.23%	95.04%	
2019	Qu Y et al. [32]	Phase II	20	Vulvar skin	Hyperspectral	75%	87.5%	/	/	/
2022	Van Vliet-Pérez S et al. [43]	Pilot	11	Ovarian cancer	Hyperspectral	81%	70%	53%	82%	/
2022	Wang P et al. [44]	Phase III	50	Cervix	Multispectral	85.3%	70.8%	/	/	/
2022	David S et al. [45]	Pilot	9	Cancer specimen from ovaries, Fallopian tubes, omentum, endometrium	Multispectral label-free Raman spectroscopy	93%	88%	/	/	90%

## Data Availability

All data generated or analyzed in this review are included in this article and/or its figures. Further inquiries may be directed to the corresponding authors.

## Supplementary Materials

The following supporting information can be downloaded at: https://www.mdpi.com/article/10.3390/diagnostics16040620/s1, File S1: Complete search strategy, Table S1: PRISMA checklist, Table S2: List of included studies, Table S3: List of excluded studies, Table S4: List of ongoing trials, Table S5: QUADAS-2 risk of bias assessment.

## Abbreviations

The following abbreviations are used in this manuscript:

AI	Artificial intelligence
CIN	Cervical intraepithelial neoplasia
CNN	Convolutional neural network
DL	Deep learning
HP-MIRSI	Hyperspectral photothermal mid-infrared spectroscopic imaging
HSI	Hyperspectral imaging
LDA	Linear discriminant analysis
ML	Machine learning
MSI	Multispectral imaging
OCT	Optical coherence tomography
RGB imaging	Red-green-blue imaging
SVM	Support vector machine
3DCNN	Three-dimensional convolutional neural network
