# Supplementary material for: From Image-Guided Surgery to Computer-Assisted Real-Time Diagnosis with Hyperspectral and Multispectral Imaging: A Systematic Review in Gynecologic Oncology
Source: Diagnostics (Basel). 2026 Feb 20;16(4):620. doi: 10.3390/diagnostics16040620 (PMC12939005; doi:10.3390/diagnostics16040620)

File S1: Search strategy

Pubmed

("Hyperspectral Imaging"[Mesh] OR "hyperspectral imaging"[tiab] OR hyperspectral[tiab]

OR "multispectral imaging"[tiab] OR multispectral[tiab]

OR "spectral imaging"[tiab] OR "optical imaging"[tiab])

AND

("Gynecology"[Mesh] OR gynecology[tiab] OR gynaecology[tiab]

OR "Gynecologic Neoplasms"[Mesh]

OR "gynecologic oncology"[tiab] OR "gynecological neoplasm*"[tiab]

OR "gynecologic cancer"[tiab])

AND (english[lang])

Google scholar and Scopus

"hyperspectral imaging" OR "multispectral imaging" OR "spectral imaging" OR "optical imaging"

"gynecologic oncology" OR "gynecological neoplasm" OR "gynecologic cancer" OR gynecology

Embase

('hyperspectral imaging'/exp OR 'hyperspectral imaging':ti,ab OR hyperspectral:ti,ab

OR 'multispectral imaging':ti,ab OR multispectral:ti,ab

OR 'spectral imaging':ti,ab OR 'optical imaging':ti,ab)

AND

('gynecology'/exp OR gynecology:ti,ab OR gynaecology:ti,ab

OR 'gynecologic tumor'/exp OR 'gynecologic cancer':ti,ab

OR 'gynecologic neoplasm*':ti,ab OR 'gynecologic oncology':ti,ab)

AND [english]/lim

ClinicalTrials.gov

(hyperspectral OR multispectral OR "spectral imaging" OR "optical imaging") AND

(gynecologic OR cervical OR ovarian OR endometrial OR uterine)

PRISMA 2020 Checklist Table S1

Section and Topic	Item #	Checklist item	Location where item is reported
**TITLE**			
Title	1	Identify the report as a systematic review.	1
**ABSTRACT**			
Abstract	2	See the PRISMA 2020 for Abstracts checklist.	1
**INTRODUCTION**			
Rationale	3	Describe the rationale for the review in the context of existing knowledge.	1-2
Objectives	4	Provide an explicit statement of the objective(s) or question(s) the review addresses.	2
**METHODS**			
Eligibility criteria	5	Specify the inclusion and exclusion criteria for the review and how studies were grouped for the syntheses.	3
Information sources	6	Specify all databases, registers, websites, organisations, reference lists and other sources searched or consulted to identify studies. Specify the date when each source was last searched or consulted.	3
Search strategy	7	Present the full search strategies for all databases, registers and websites, including any filters and limits used.	3, S1
Selection process	8	Specify the methods used to decide whether a study met the inclusion criteria of the review, including how many reviewers screened each record and each report retrieved, whether they worked independently, and if applicable, details of automation tools used in the process.	3
Data collection process	9	Specify the methods used to collect data from reports, including how many reviewers collected data from each report, whether they worked independently, any processes for obtaining or confirming data from study investigators, and if applicable, details of automation tools used in the process.	3
Data items	10a	List and define all outcomes for which data were sought. Specify whether all results that were compatible with each outcome domain in each study were sought (e.g. for all measures, time points, analyses), and if not, the methods used to decide which results to collect.	3
	10b	List and define all other variables for which data were sought (e.g. participant and intervention characteristics, funding sources). Describe any assumptions made about any missing or unclear information.	3
Study risk of bias assessment	11	Specify the methods used to assess risk of bias in the included studies, including details of the tool(s) used, how many reviewers assessed each study and whether they worked independently, and if applicable, details of automation tools used in the process.	3
Effect measures	12	Specify for each outcome the effect measure(s) (e.g. risk ratio, mean difference) used in the synthesis or presentation of results.	3
Synthesis methods	13a	Describe the processes used to decide which studies were eligible for each synthesis (e.g. tabulating the study intervention characteristics and comparing against the planned groups for each synthesis (item #5)).	3
	13b	Describe any methods required to prepare the data for presentation or synthesis, such as handling of missing summary statistics, or data conversions.	3
	13c	Describe any methods used to tabulate or visually display results of individual studies and syntheses.	3-4
	13d	Describe any methods used to synthesize results and provide a rationale for the choice(s). If meta-analysis was performed, describe the model(s), method(s) to identify the presence and extent of statistical heterogeneity, and software package(s) used.	3
	13e	Describe any methods used to explore possible causes of heterogeneity among study results (e.g. subgroup analysis, meta-regression).	3
	13f	Describe any sensitivity analyses conducted to assess robustness of the synthesized results.	3
Reporting bias	14	Describe any methods used to assess risk of bias due to missing results in a synthesis (arising from reporting biases).	3

Section and Topic	Item #	Checklist item	Location where item is reported
assessment			
Certainty assessment	15	Describe any methods used to assess certainty (or confidence) in the body of evidence for an outcome.	3
**RESULTS**			
Study selection	16a	Describe the results of the search and selection process, from the number of records identified in the search to the number of studies included in the review, ideally using a flow diagram.	5
	16b	Cite studies that might appear to meet the inclusion criteria, but which were excluded, and explain why they were excluded.	5
Study characteristics	17	Cite each included study and present its characteristics.	5-9
Risk of bias in studies	18	Present assessments of risk of bias for each included study.	S6
Results of individual studies	19	For all outcomes, present, for each study: (a) summary statistics for each group (where appropriate) and (b) an effect estimate and its precision (e.g. confidence/credible interval), ideally using structured tables or plots.	5-9
Results of syntheses	20a	For each synthesis, briefly summarise the characteristics and risk of bias among contributing studies.	5-9
	20b	Present results of all statistical syntheses conducted. If meta-analysis was done, present for each the summary estimate and its precision (e.g. confidence/credible interval) and measures of statistical heterogeneity. If comparing groups, describe the direction of the effect.	5-9
	20c	Present results of all investigations of possible causes of heterogeneity among study results.	9
	20d	Present results of all sensitivity analyses conducted to assess the robustness of the synthesized results.	5-9
Reporting biases	21	Present assessments of risk of bias due to missing results (arising from reporting biases) for each synthesis assessed.	S6
Certainty of evidence	22	Present assessments of certainty (or confidence) in the body of evidence for each outcome assessed.	5-9
**DISCUSSION**			
Discussion	23a	Provide a general interpretation of the results in the context of other evidence.	9-13
	23b	Discuss any limitations of the evidence included in the review.	13-14
	23c	Discuss any limitations of the review processes used.	13-14
	23d	Discuss implications of the results for practice, policy, and future research.	14
**OTHER INFORMATION**			
Registration and protocol	24a	Provide registration information for the review, including register name and registration number, or state that the review was not registered.	2-3
	24b	Indicate where the review protocol can be accessed, or state that a protocol was not prepared.	3
	24c	Describe and explain any amendments to information provided at registration or in the protocol.	15
Support	25	Describe sources of financial or non-financial support for the review, and the role of the funders or sponsors in the review.	15
Competing interests	26	Declare any competing interests of review authors.	15

PRISMA 2020 Checklist Table S1

Section and Topic	Item #	Checklist item	Location where item is reported
Availability of data, code and other materials	27	Report which of the following are publicly available and where they can be found: template data collection forms; data extracted from included studies; data used for all analyses; analytic code; any other materials used in the review.	15

From: Page MJ, McKenzie JE, Bossuyt PM, Boutron I, Hoffmann TC, Mulrow CD, et al. The PRISMA 2020 statement: an updated guideline for reporting systematic reviews. BMJ 2021;372:n71. doi: 10.1136/bmj.n71. This work is licensed under CC BY 4.0. To view a copy of this license, visit https://creativecommons.org/licenses/by/4.0/

Table S2

YEAR	AUTHOR	COUNTRY	TITLE	STUDY DESIGN	IDEAL stage	SAMPLE SIZE	TARGET TISSUE	OPTICAL TYPE	CAMERA TYPE	SCAN METHOD (spatial spectral, snapshot)	SPECTRAL RANGE (nm)	RESOLUTION	ACQUISITION TIME	*IN VIVO / EX VIVO*	AIM	GOLD STANDARD	AI MODEL
2000	Parker MF et al.	United States	Hyperspectral diagnostic imaging of the cervix: report on a new investigational device.	Prospective	1	35	Cervix	Hyperspectral	/	Spatial	400-800 nm	/	/	*in vivo*	Initial experience with HSI for the detection and localization of cervical intraepithelial neoplasia (CIN).	Colposcopy, cytology, histopathology	Not performed
2001	Ferris DG et al.	United States	Multimodal hyperspectral imaging for the noninvasive diagnosis of cervical neoplasia.	Prospective	2b	111	Cervix	Hypespectral	LuViva®, Guided Therapeutics, Inc, Norcross, GA	Snapshot	300-500 nm	/	/	*in vivo*	Multimodal Hyperspectral Imaging for no ninvasive detection, localization and diagnosis of cervical neoplasia.	Colposcopy, cytology, histopathology	Machine learning (Logistic regression model, linear discriminant analysis)
2001	Balas C	Greece	A Novel Optical Imaging Method for the Early Detection, Quantitative Grading, and Mapping of Cancerous and Precancerous Lesions of Cervix.	Prospective	1	16	Cervix	Multispectral	/	/	400-700 nm	/	/	*in vivo*	Multispectral imaging system for *in vivo* early detection, quantitative staging, and mapping of cervical cancer and precancerous lesions.	Histopathology	Not perfomed
2002	Parker MF et al.	United States	Initial neural net construction for the detection of cervical intraepithelial neoplasia by fluorescence imaging.	Prospective	2a	33	Cervix	Hyperspectral	/	/	401-770 nm	/	/	*in vivo*	Integration of neural networks for the detection of cervical intraepithelial neoplasia by fluorescence imaging with a hyperspectral optical imaging device.	Cytology, histopathology	Deep learning (Neural network)
2003	Benavides J et al.	United States	Multispectral digital colposcopy for in vivo detection of cervical cancer.	Prospective	2a	146	Cervix	Multispectral	CV S3200, JAI, Japan	Snapshot	330-440 nm	8 by 8 µm pixels	5-10 s	*in vivo*	Evaluate the application of *in vivo* multispectral digital colposcopy in detecting cervical intraepithelial neoplasia.	Histopathology	Machine learning (not specified)
2005	Milbourne A et al.	United States	Results of a pilot study of multispectral digital colposcopy for the in vivo detection of cervical intraepithelial neoplasia.	Prospective	2b	46	Cervix	Multispectral	CV S3200, JAI, Japan	Snapshot	345-440 nm	/	< 1 min	*in vivo*	Pilot study of multispectral digital colposcopy for in vivo detection of cervical intraepithelial neoplasia.	Colposcopy, histopathology	Machine learning (k means clustering and k nearest)
2005	Orfanoudaki IM et al.	Greece	A clinical study of optical biopsy of the uterine cervix using a multispectral imaging system.	Prospective	2b	134	Cervix	Multispectral	FO.R.T.H Instruments, Heraklion, GR	/	500-550 nm	/	/	*in vivo*	Evaluate the clinical application of *in vivo* multispectral imaging colposcopic system.	Colposcopy, cytology, histopathology	Not performed
2007	DeSantis T et al.	United States	Spectroscopic imaging as a triage test for cervical disease: a prospective multicenter clinical trial.	Prospective	2b	572	Cervix	Hyperspectral	LuViva®, Guided Therapeutics, Inc, Norcross, GA	Snapshot	300-500 nm	/	/	*in vivo*	Spectroscopic imaging for the diagnosis of cervical cancer in a prospective multicenter study.	Colposcopy, cytology, histopathology	Not performed
2008	Park SY et al.	United States	Automated image analysis of digital colposcopy for the detection of cervical neoplasia.	Prospective	2b	29	Cervix	Multispectral	CV S3200, JAI, Japan	Snapshot	/	/	/	*in vivo*	Pilot study on multispectral digital colposcope for identification of neoplastic tissue from digital images.	Histopathology	Machine learning (Ensemble classifier)
2011	van Dam GM et al.	Netherlands	Intraoperative tumor-specific fluorescence imaging in ovarian cancer by folate receptor-α targeting: first in-human results.	Prospective	2a	10	Ovarian tissue and carcinomatosis specimens	Multispectral fluorescence imaging	Technical University Munich/Helmholtz Center Munich (by G.T., A.S. and V.N.	/	495 - 520 nm	150 to 30 µm	/	*in vivo and ex vivo*	First-in-human use of intraoperative tumor-specific fluorescence imaging for real-time surgical visualization of tumor tissue in patients undergoing an exploratory laparotomy for suspected ovarian cancer.	Histopathology	Not performed
2012	Renkoski TE et al.	United States	Wide-field spectral imaging of human ovary autofluorescence and oncologic diagnosis via previously collected probe data.	Prospective	2a	30	Ovaries	Multispectral	SEAtreat, Apogen Technologies, Inc., now QinetiQ North America, San Diego, CA	/	400-640 nm	0.06 mm/pixel	5-8 min	*ex vivo*	Evaluate multispectral imaging and autofluorescence spectroscopy in detecting ovarian cancer.	Histopathology	Machine learning (Linear discriminant analysis)
2013	Kavvadias V et al.	Japan	A novel endoscopic spectral imaging platform integrating k-means clustering for early and non-invasive diagnosis of endometrial pathology.	Prospective	1	15	Endometrium	Multispectral	color camera Sony ICX674	Snapshot	400-1000 nm	/	/	*in vivo*	Pilot study about multispectral imaging system and machine learning algorithm in vivo analysis of the endometrium.	Histopathology	Machine learning (K means clustering)
2013	Twiggs LB et al.	United States	Multimodal hyperspectroscopy as a triage test for cervical neoplasia: Pivotal clinical trial results.	Prospective	2b	1607	Cervix	Multimodal hyperspectral imaging	LuViva®, Guided Therapeutics, Inc, Norcross, GA	Snapshot	300-500 nm	/	1-4.5 min	*in vivo*	Provide a prospective evaluation of the sensitivity and specificity of multispectral imaging for the detection of moderate and high grade dysplasia.	Histopathology	Not performed
2015	Zheng W et al.	United States	Hyperspectral wide gap second derivative analysis for in vivo detection of cervical intraepithelial neoplasia.	Prospective	2a	3	Cervix	Hyperspectral	AOTF hyperspectral imager (Brimrose, Sparks, Maryland)	Spectral	600-800 nm	1392 × 1040 pixel, 6.45-µm pixel size	10 s	*in vivo*	Propose an image processing procedure to classify cervical tissue into normal, inflammation and high-grade lesion (CIN 2 or 3) types by wide gap second derivative spectral analysis and image segmentation.	Histopathology	Not performed
2016	Qu Y et al.	United States, China	Polarized hyperspectral imaging system for in vivo detection of vulvar lichen sclerosis.	Prospective	1	/	Vulvar skin	Hyperspectral	Brimrose Hyperspectral Imager (Brimrose, Sparks, Maryland)	Spatial	450-650 nm	/	10 s	*in vivo*	Develope a polarized, hyperspectral imaging system for in vivo quantitative assessment of vulvar lichen sclerosis	Histopathology	Not perfomed
2016	Tate TH et al.	United States	Multispectral fluorescence imaging of human ovarian and fallopian tube tissue for early-stage cancer detection.	Prospective	2a	28	Ovaries	Multispectral fluorescence imaging	CCD (PhotonMAX: 512B, Princeton Instruments, Trenton, New Jersey)	Snapshot	260-650 nm	/	/	*ex vivo*	Multispectral fluorescence imaging for ovarian cancer detection	Histopathology	Not perfomed
2018	Nandy S et al.	United States	Evaluation of Ovarian Cancer: Initial Application of Coregistered Photoacoustic Tomography and US.	Prospective	2a	16	Ovaries	Multispectral photoacoustic imaging	Ti-sapphire laser (Symphotics, Camarillo, Calif)	/	690–900 nm	/	/	*in vivo*	Pilot study to evaluate multispectral photoacustic imaging in distinguishing benign from malignant ovarian tumors.	Histopathology	Not perfomed
2018	Nandy S et al.	United States	Quantitative multispectral ex vivo optical evaluation of human ovarian tissue using spatial frequency domain imaging.	Prospective	2a	11	Ovaries	Multispectral	CCD camera Basler ace, 30 fps, dynamic range 57 dB	Snapshot	460-630 nm	/	/	*ex vivo*	Multispectral *ex vivo* imaging for quantitative, wide-field evaluation of freshly excised benign and malignant human ovarian tissues	Histopathology	Machine learning (Logistic regression model)
2019	Qu Y et al.	United States, China	Therapeutic assessment of high-intensity focused ultrasound for vulvar lichen sclerosus by active dynamic thermal imaging and hyperspectral imaging.	Prospective	2a	20	Vulvar skin	Hyperspectral	Brimrose Hyperspectral Imager (Brimrose, Sparks, Maryland)	Spatial	450 - 650 nm	/	10 s	*in vivo*	Investigate the feasibility of HSI for quick non-invasive assessment of the therapeutic response to HIFU in VLS based on the changes caused in tissue properties.	Clinical score assessed by the gynecologist (cured, effective, ineffective)	Machine learning (Linear discriminant analysis)
2020	Yu W et al.	China	High resolution multispectral endoscopy significantly improves the diagnostic accuracy of cervical intraepithelial lesions.	Prospective	2a	11	Cervix	Multispectral	cam1530, Opto-Medic, Guangdong, China	/	420- 525 nm	1920 × 1080 pixel 60 frames	/	*in vivo*	Describe the performance of high-resolution multispectral endoscopy in cervical lesion detection	Colposcopy, cytology, histopathology	Not perfomed
2022	Van Vliet-Pérez S et al.	Switzerland	Hyperspectral Imaging for Tissue Classification after Advanced Stage Ovarian Cancer Surgery-A Pilot Study.	Prospective	2a	11	Ovarian tissue and carcinomatosis specimens	Hyperspectral	IMEC, Snapscan NIR camera (Leuven, Belgium)	Snapshot	665–975 nm	2050 x 1080 pixel	/	*ex vivo*	Evaluate the feasibility of HSI and machine learning model for EOC detection in ex vivo tissue samples.	Histopathology	Machine learning (Support vector machine classifier)
2022	Wang P et al.	China	Multispectral Image under Tissue Classification Algorithm in Screening of Cervical Cancer.	Prospective	2b	50	Cervix	Multispectral	/	/	/	/	/	*in vivo*	Explore the application of tissue classification algorithm combined with multispectral imaging in screening of cervical cancer.	Histopathology	Machine learning (K means clustering)
2022	David S et al.	Canada	Multispectral label-free Raman spectroscopy can detect ovarian and endometrial cancer with high accuracy.	Prospective	2a	9	Cancer specimens from ovaries, fallopian tubes, omentum and endometrium	Multispectral Label-Free Raman Spectroscopy	Innovative Photonic Solutions, New Jersey	/	785 nm	/	/	*ex vivo*	Multispectral label-free Raman spectroscopy for the detecion of ovarian and endometrial cancer.		Machine learning (Support vector machine classifier)
2023	Schimunek L et al.	Germany	Hyperspectral imaging as a new diagnostic tool for cervical intraepithelial neoplasia.	Prospective	2b	41	Cervix	Hypespectral	TIVITA® Tissue device (Dia spective Vision GmbH, Pepelow, Germany).	Snapshot	500–995 nm	0.45 mm/pixel	/	*in vivo*	Evaluate the feasibility of a commercially available HSI system for CIN differentiation in a prospective monocentric clinical trial.	Histopathology	Not perfomed
2023	Rocha AD et al.	United States	Iterative prototyping based on lessons learned from the falloposcope in vivo pilot study experience.	Prospective	1	12	Fallopian tubes	Multispectral fluorescence imaging, OCT	Teledyne Photometrics, Retiga R6 camera	Snapshot	405-642 nm	11 x 17 µm	/	*in vivo*	Pilot study to develop an FT endoscope, the falloposcope, as a method for FTs visualization. The study was performed on volunteers with no evidence of or elevated risk for ovarian cancer.	/	Not perfomed
2024	Reihanisaransari R et al.	United States	Cervical Cancer Tissue Analysis Using Photothermal Midinfrared Spectroscopic Imaging.	Prospective	1	98	Cervix	Hyperspectral photothermal mid-infrared spectro scopic imaging (HP-MIRSI)	/	/	974-1786 nm	/	80 min per band at high spatial resolution and 8 min per band at the lower spatial resolution	*ex vivo*	Application of HS-MIRSI and machine learning method, for tissue subtype classification of cervical cancer.	Histopathology	Machine learning (Random forest model) and deep learning (Convolutional neural network)
2024	Karthika J et al.	Germany	Label-free assessment of the transformation zone using multispectral diffuse optical imaging toward early detection of cervical cancer.	Prospective	2a	5	Cervix	Multispectral	GynoSight v1.0 (GC2755 imaging sensor Y002 Supereyes camera)	Snapshot	450-620 nm	10.08 lines/mm	17 s	*in vivo*	Pilot study to design and develop a portable, affordable, and label-free transvaginal imaging probe (GynoSight v1.0) to acquire multispectral images from the cervix and assess the suspicious region using spectral contrast ratio and the assessment of the TZ .	Colposcopy, cytology, histopathology	Not perfomed
2025	Vega C et al.	Spain	Feasibility study of hyperspectral colposcopy as a novel tool for detecting precancerous cervical lesions.	Prospective	2a	62	Cervix	Hyperspectral	IMEC Snapscan VNIR camera (Leuven, Belgium) coupled to the Optomic OPC2 colposcope (OPTOMIC, Spain)	Snapshot	470-900 nm	14.7 pixels/mm	45 s	*in vivo*	Assess the feasibility of HSI for in-vivo analysis of cervical lesions.	Colposcopy, cytology	Not performed
2025	Rocha AD et al.	United States	First Clinical Feasibility and Safety Study of a Novel Multimodality Fallopian Tube Imaging Endoscope.	Prospective	1	19	Fallopian tubes	Multispectral, OCT	/	/	405-642 nm	11 x 17 µm	/	*in vivo and ex vivo*	Assess the safety and feasibility of using optical imaging falloposcope to visualize the FTs, assess any damage caused to the FTs by the falloposcope, and to obtain qualitative physician feedback on the operation of the falloposcope and the quality of the images.	Histopathology	Not perfomed

Table S3: Excluded articles

Year	Authors	Article	Exclusion reasons	PMID
2019	St-George Robillard A	Long-term fluorescence hyperspectral imaging of on-chip treated co-culture tumour spheroids to follow clonal evolution	Wrong study design	31172192
2024	Reihanisaransari R	Rapid hyperspectral photothermal mid-infrared spectroscopic imaging from sparse data for gynecologic cancer tissue subtyping	Wrong study type	38463509
2011	Orfanoudaki I	Recent advances in optical imaging for cervical cancer detection	Wrong study type	21800084
2019	David A	Current advances in optical screening for cervical cancer	Wrong study type	-
2018	Paiman AP	Advancing In-vivo Vulvar Tumour Detection: Combining AI and Hyperspectral Imaging	Wrong study type	-
2024	Marti Garcia D	Hyperspectral imaging as a potentially non-invasive method allowing discrimination of endometrial menstrual cycle phases and identification of the window of implantation in normo-ovulatory women	Wrong population	-
2018	Saso S	Use of biomedical photonics in gynecological surgery: a uterine transplantation model.	Wrong study design	29682321
2022	Rehbinder	Depolarization imaging for fast and non-invasive monitoring of cervical microstructure remodeling in vivo during pregnancy.	Wrong populaiton	-
2020	Dutta R	Brilliant cresyl blue enhanced optoacoustic	Wrong population	33143591

		imaging enables non-destructive imaging of mammalian ovarian follicles for artificial reproduction		
2018	Samykutty A	Optoacoustic imaging identifies ovarian cancer using a microenvironment targeted theranostic wormhole mesoporous silica nanoparticle	Wrong study design	30118979
2016	Lewellen A	Quantitation of Intra-peritoneal Ovarian Cancer Metastasis	Wrong study design	27500635
2010	Crane L	Multispectral real-time fluorescence imaging for intraoperative detection of the sentinel lymph node in gynecologic oncology	Wrong study design	21048667
2011	Crane L	Intraoperative multispectral fluorescence imaging for the detection of the sentinel lymph node in cervical cancer: a novel concept	Wrong study design	20835767

Table S4: Ongoing clinical trials *(abbreviations: LEEP, loop electrosurgical excision procedure).*

Registry Number	Study Title	Sample size	Country	Condition/Disease	Study design	Intervention/Treatment	Primary outcome measure	Recruitment state
NCT02406352	Multispectral Digital Colposcope With Probe for Detection of Cervical Intraepithelial Neoplasia (MDCwProbe)	618	United States	Patients referred for colposcopy or LEEP treatment	Prospective	Device: Multispectral digital colposcope	Detection of precancerous cervical lesions or cervical cancer using multispectral imaging	N/A
NCT04915495	The Use of the LuViva Advanced Cervical Scan to Identify Women at High-Risk for Cervical Neoplasia	400	United States	Patients scheduled for colposcopy	Prospective	Device: Multimodal hyperspectral imaging	Sensitivity and specificity of the diagnostic device (LuViva Advanced Cervical Scan)	Enrolling by invitation

Table S5: QUADAS-2 risk of bias graphical representation.

Year	Study	RISK OF BIAS				APPLICABILITY CONCERNS		
		PATIENT SELECTION	INDEX TEST	REFERENCE STANDARD	FLOW AND TIMING	PATIENT SELECTION	INDEX TEST	REFERENCE STANDARD
2000	Parker MF	☹	☹	?	☹	☹	☹	☺
2001	Ferris DG	☺	?	☹	☹	☹	☹	?
2001	Balas C	?	?	☹	☹	☹	☹	☹
2002	Parker MF	?	☹	?	☹	☹	☹	☹
2003	Benavides J	☹	☹	?	?	☹	☹	☺
2005	Milbourne A	?	☹	?	?	☹	☹	☺
2005	Orfanoudaki IM	☹	☹	?	?	?	☹	☹
2007	DeSantis T	?	☹	☹	☹	?	☹	☹
2008	Park SY	?	☹	?	☺	☹	☹	☺
2011	van Dam GM	☺	☺	?	☺	?	☺	☺
2012	Renkoski TE	?	☺	☹	?	?	?	☹
2013	Kavvadias V	?	☹	?	?	☹	?	☹
2013	Twiggs Leo B	☺	?	☺	☺	☺	☺	☺
2015	Zheng W	?	☹	?	☹	☹	?	☹
2016	Qu Y	?	☹	?	?	☹	☹	☺
2016	Tate TH	?	☹	☺	☺	?	☹	☺
2018	Nandy S	☹	?	?	☺	☹	?	?
2018	Nandy S	☹	☹	?	☺	☹	☹	☺
2022	Van Vliet-Pérez S	?	?	☺	☺	☹	☹	☺
2022	Wang P	☹	☹	☹	☹	☹	☹	☹
2022	David S	☹	?	☺	☺	☹	☹	☺
2023	Schimunek L	☹	☹	?	☺	☹	☹	☺
2024	Karthika J	☹	☹	☺	?	☹	☹	☺
2024	Reihanisaransari R	☹	☺	☺	☺	☹	☺	☺
2025	Vega C	?	☹	☺	?	?	☹	☺

☺ Low Risk ☹ High Risk ? Unclear Risk

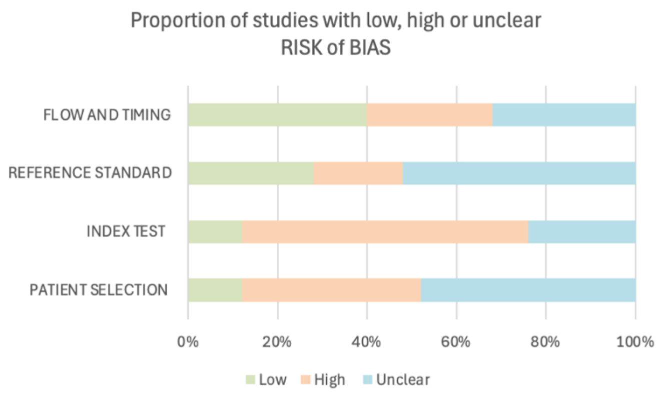

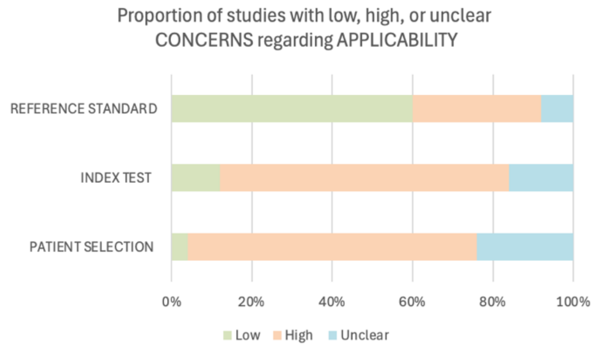

Supplement: Supplementary file 1 [file diagnostics-16-00620-s001.zip › diagnostics-4087733-supplementary.pdf]
